# Le syndrome de Pepper: à propos de deux cas observés au Centre Hospitalier Universitaire Pédiatrique Charles de Gaulle de Ouagadougou (Burkina Faso)

**DOI:** 10.11604/pamj.2017.28.189.11901

**Published:** 2017-10-31

**Authors:** Madibèlè Kam, Sonia Douamba, Kisito Nagalo, Lassina Dao, Fla Kouéta, Claudine Lougué, Diarra Yé

**Affiliations:** 1 Service de Pédiatrie Médicale, CHU Pédiatrique Charles de Gaulle, Ouagadougou, Burkina Faso; 2 UFR SDS, Université Ouaga I Pr. Joseph Ki-Zerbo, Ouagadougou, Burkina Faso; 3 Service de Radiologie, CHU Pédiatrique Charles de Gaulle, Ouagadougou, Burkina Faso

**Keywords:** Neuroblastome, syndrome de Pepper, enfant, Neuroblastoma, Pepper’s syndrome, infant

## Abstract

Le syndrome de Pepper est une forme métastatique hépatique du neuroblastome. C’est une entité spécifique du nourrisson de moins de six mois qui a la particularité de pouvoir régresser de façon spontanée avec un pronostic favorable dans 80% des cas. A cause de sa rareté, nous rapportons deux cas du syndrome de Pepper observés au Centre Hospitalier Universitaire Pédiatrique Charles de Gaulle de Ouagadougou (Burkina Faso). Il s’agissait de deux nourrissons de sexe féminin chez qui la symptomatologie de la maladie se traduisait par une augmentation du volume abdominal, une hépatomégalie et des signes de lutte respiratoire. L'échographie a permis de poser le diagnostic par l’aspect nodulaire du foie dans les deux cas et la détermination de la tumeur primitive dans un cas. Le dosage des catécholamines urinaires a confirmé un cas. L’évolution était fatale chez les deux patientes du fait des complications de compression par le foie, des complications de la chimiothérapie dans l’un des cas et l’absence de traitement dans l’autre cas.

## Abstract (en)

Pepper’s syndrome is a neuroblastoma that metastasizes to the liver. It affects infants younger than six months of age. It can regress spontaneously and is associated with a favorable prognosis in 80% of cases. Given its rarity, we here report two cases of Pepper’s syndrome observed at the Charles de Gaulle university pediatric hospital center, Ouagadougou (Burkina Faso). Our study involved two female infants in whom the disease manifested as an increase in abdominal volume, hepatomegaly and signs of respiratory distress. Ultrasound enabled diagnosis, which was based on the nodular appearance of the liver in both cases and determination of the primary tumor in one case. Urinary catecholamine test confirmed the diagnosis in one case. Both patients died from complications related to liver compression, chemotherapy (in one of the cases) and lack of treatment (in the other case).

## Introduction

Le syndrome de Pepper est un envahissement métastatique du foie par un neuroblastome le plus souvent d'origine surrénalienne. Cette forme clinique particulière du neuroblastome métastatique touche principalement le nourrisson avant l’âge d'un an [1]. Malgré la malignité du neuroblastome, le syndrome de Pepper reste de bon pronostic à long terme avec une possible régression spontanée; on note seulement 10 à 20% de décès précoces suite à des complications [1]. Il s’agit d’une entité spécifique du nourrisson de moins de six mois, le seul exemple en oncologie où l’on peut assister à une régression spontanée et qui n’impose pas un traitement systématique [2]. Le syndrome de Pepper est rare, on dénombre environ 15 cas/an en France [3]. Au Burkina Faso, la fréquence n'est pas connue. Le but de ce travail était de rapporter deux cas du syndrome de Pepper observés dans le service de pédiatrie du Centre Hospitalier Universitaire Pédiatrique Charles de Gaulle (CHUP-CDG) de Ouagadougou.

## Patient et observation

### Observation N°1

Ce nourrisson de sexe féminin, âgé de un mois et demi était admise aux urgences médicales pour augmentation du volume de l’abdomen. La grossesse et l'accouchement étaient normaux. Elle était le sixième enfant d’une fratrie de six enfants; ses aînés étaient en bon état de santé apparent. Ses parents, qui ne sont pas consanguins, ont constaté une augmentation progressive du volume de son abdomen malgré l'administration de trimébutine orale. A Jour 5 d'hospitalisation, l'enfant est transférée en réanimation pour une détresse respiratoire. L'examen général notait un fébricule à 37,7°C, un mauvais état général, des œdèmes des membres inférieurs, une pâleur conjonctivale, une polypnée à 66 c/min, un regard en coucher de soleil. L’examen physique montrait un abdomen volumineux, luisant avec circulation veineuse collatérale, difficilement dépressible avec une hépatomégalie (flèche hépatique à 20 cm) ferme à surface lisse et bord inferieur mousse qui occupait l’hypochondre droit et descendait jusqu’à la fosse iliaque droite. On notait une splénomégalie stade 4 de Hackett. Sur le plan biologique, il y avait une anémie sévère (taux d’hémoglobine à 5,5 g/dl), une thrombopénie (plaquettes à 82 000/mm^3^), un taux de prothrombine de 12%, un temps de céphaline activé allongé à 52 secondes. L’échographie abdominale montrait un foie hétérogène avec de nombreux nodules et une masse rétropéritonéale gauche qui évoquaient un neuroblastome. Les catécholamines urinaires n’étaient pas être réalisées. Le traitement était fait d'une transfusion de concentré de globules rouges, de fer, de vitamine K, d'antipaludique (artésunate/luméfantrine), d'antibiotique (ceftriaxone et gentamycine). Les parents ont bénéficié d’une prise en charge psychologique. En l’absence de traitement curatif possible, l'enfant est décédée après quatre semaines d’hospitalisation par compression abdominale secondaire à l’importante hépatomégalie.

### Observation N° 2

Ce nourrisson âgé de trois mois, de sexe féminin, était admis pour distension abdominale évoluant depuis la naissance et pâleur associée. Dans les antécédents, la grossesse s'est bien déroulée et l'accouchement était normal à terme. L'interrogatoire notait de multiples consultations pour ballonnement abdominal traité au trimébutine sans succès. L’examen à l'admission retrouvait un état général médiocre, une pâleur palmo-plantaire et conjonctivale, une détresse respiratoire modérée, un abdomen volumineux et tendu avec une circulation veineuse collatérale, une hépatomégalie ferme, sensible, occupant l’hypochondre droit, le flanc droit et la fosse iliaque droite et s’étendant jusqu’au flanc et à la fosse iliaque gauches. Sur le plan biologique, on notait une anémie (taux d’hémoglobine à 6 g/dl), une leucocytose à 12200/mm^3^, des plaquettes à 113000/mm^3^. Les transaminases hépatiques, la bilirubinémie totale étaient normales, le taux de prothrombine était diminué à 34%, les LDH augmentés à 1746 UI/l, l’alpha fœto-protéine augmentée à 700 UI/ml, les catécholamines urinaires augmentées avec un rapport VMA/créatinine à 53,38 μmol/mmol et HVA/créatinine à 223,53μmol/mmol. L’échographie abdominale montrait une hépatomégalie hétérogène associée à une masse surrénalienne gauche avec un centre liquidien faisant évoquer un syndrome de Pepper. La tomodensitométrie abdominale retrouvait une hépatomégalie pluri nodulaire et une tumeur surrénalienne gauche. L’évolution était marquée par une augmentation progressive du volume de l’abdomen (le périmètre abdominal est passé de 45 cm à 56 cm en 12 jours) qui a eu pour effet d'aggraver la détresse respiratoire et majorer la circulation veineuse collatérale abdominale. Deux cures d'une chimiothérapie anticancéreuse étaient faites avec une combinaison carboplatine 18 mg/kg/j+étoposide 4mg/kg/j. Au décours de cette chimiothérapie, survenaient un syndrome infectieux, une diarrhée aqueuse et un syndrome hémorragique constitué par une dysenterie et une épistaxis de moyenne abondance associées à une thrombopénie. Un tableau d’aplasie fébrile compliquait l’évolution et entraînait le décès de l'enfant par arrêt cardiorespiratoire après un mois et demi d’hospitalisation.

## Discussion

Le syndrome de Pepper est une maladie rare; on en dénombre environ 15 cas par an en France [3]. A ce jour, à notre connaissance, il n'y avait aucun cas publié au Burkina Faso, sans doute du fait de cette rareté mais aussi de sa méconnaissance et/ou de sous diagnostic dans nos conditions de travail d'un pays en développement. L'âge des deux patients dans notre étude montre bien que le syndrome de Pepper est une entité spécifique surtout du nourrisson [1, 3, 4]. Si le neuroblastome touche de manière égale les deux sexes [3], notre étude montre un sex-ratio en faveur du sexe féminin dans le syndrome de Pepper en adéquation avec d'autres études [5].

Sur le plan clinique, l'hépatomégalie retrouvée dans nos deux observations montre bien qu'elle constitue la circonstance de découverte du syndrome de Pepper [1, 6]. Dans les deux cas présentés, le ballonnement abdominal était au premier plan, à l'origine d'une possible errance diagnostique du fait d’un symptôme banal. Il semble important de rechercher une hépatomégalie devant tout météorisme abdominal et suspecter un syndrome de Pepper devant toute hépatomégalie progressive du nourrisson. Cette difficulté diagnostique est aussi reconnue dans la littérature [7]. La diarrhée décrite dans nos observations pourrait rentrer dans le cadre d’une diarrhée motrice par sécrétion de Vasoactive Intestinale Peptide (VIP) telle que rapportée chez 20 % des patients présentant un neuroblastome [3].

Sur le plan biologique, le syndrome de Pepper, bien que comportant une atteinte métastatique du foie, montre habituellement un bilan hépatique normal [7]. Cependant, nous avons noté une baisse du taux de prothrombine dans nos deux cas, ce qui n’est pas habituel. La positivité des catécholamines urinaires était un élément de certitude diagnostique dans l'observation n° 2, en conformité avec ce qui est rapporté dans la littérature [3]. La contribution de l’imagerie au diagnostic dans notre étude permet de confirmer la place capitale de l'échographie et du scanner abdominal dans le diagnostic du syndrome de Pepper [7-9]. Ce sont deux examens complémentaires non invasifs qui sont accessibles même aux pays en développement.

L'évolution était défavorable dans les deux cas que nous rapportons. Cette forme d’évolution rapide qui est également rapportée par d'autres auteurs semble être fréquente dans le syndrome de Pepper. Les complications à type de détresse respiratoire par compression due à l'hépatomégalie observées dans nos cas sont également rapportées [1, 5, 6, 10]. L'un des éléments de mauvais pronostic était un rapport initial VMA/HVA < 1,5 car certains auteurs ont rapporté une évolution plus favorable lorsque le rapport initial VMA/HVA est supérieur à 1,5 [11], or dans l'observation N° 2 ce rapport était de 0,23. Le deuxième élément de mauvais pronostic était l'élévation du LDH (LDH à 1746 UI/l dans l'observation n° 2). En effet, il est rapporté que les enfants porteurs d’un neuroblastome avec LDH augmentée ont une survie de 20 % alors que les autres ont une probabilité de survie de 80 % [11]. Le retard au diagnostic et au traitement ont contribué au décès des deux nourrissons dans notre étude. D’autres éléments pronostiques tels que l’antigénémie Nmyc sur la tumeur et la diploïdie n’étaient pas recherchées dans nos deux cas par manque de moyens financiers et d'un laboratoire local performant.

## Conclusion

Le syndrome de Pepper est une pathologie tumorale du nourrisson qui est rare et ubiquitaire. Les deux cas que nous rapportons illustrent la difficulté du diagnostic et du traitement que le syndrome pose dans les pays à faible revenu.

## Conflits d’intérêts

Les auteurs ne déclarent aucun conflit d’intérêts.
